# Histopathological changes in the human tissues in various types of poisoning: A cross-sectional autopsy study

**DOI:** 10.1016/j.toxrep.2024.101771

**Published:** 2024-10-15

**Authors:** Jayeshkumar Kanani, Mohammed Iliyas Sheikh, Sudha Jain, Swati Mesuriya

**Affiliations:** a Dept. of Forensic Medicine and Toxicology, Surat Municipal Institute of Medical Education and Research, India; b Dept. of Pathology, Surat Municipal Institute of Medical Education and Research, India

**Keywords:** Poisoning, Organophosphates, Aluminum phosphide, Histopathological change, Autopsy, Pesticides, Suicide, Toxicology, Viscera, Corrosive poisons

## Abstract

**Background:**

Poisoning is a critical health issue caused by exposure to harmful substances, leading to a range of biological effects from mild irritation to severe organ damage and death. Acute poisoning is particularly prevalent in developing countries reliant on agriculture, where agricultural poisons such as organophosphates, carbamates, pyrethroids, and aluminum/zinc phosphide are common. This study aims to analyze the histopathological changes in various organs in autopsy of poisoning cases to understand the extent and nature of organ damage.

**Methods:**

Autopsies were performed on cases with an established or suspected history of poison ingestion. Tissue samples from the stomach, intestine, liver, spleen, kidneys, brain, and lungs were examined for histopathological changes.

**Results:**

Out of 52 cases analyzed, aluminum phosphide was the predominant poison, accounting for 76.92 % of cases. Histopathological findings in poisoning cases included significant pulmonary edema (55.77 %), intra-alveolar hemorrhage (48.08 %), liver ballooning degeneration (48.08 %), acute tubular necrosis (51.92 %) in kidney, and universal brain and spleen congestion (100 %). Stomach findings showed partial loss of rugosity (80.77 %), congestion (51.92 %), necrosis of the mucosa (30.77 %), congestion (71.15 %), denudation of the epithelium (48.08 %), and mucosal inflammation (48.08 %) as predominant findings. Aluminum phosphide caused severe histopathological changes across all examined organs.

**Conclusion:**

The study highlights the critical role of histopathological examination in diagnosing and understanding organ damage in poisoning cases. Forensic pathologists can use these histopathological patterns as reference points to differentiate poisoning from other causes of death, aiding in accurate diagnosis and targeted treatment.

## Highlights

• Aluminum phosphide was identified as the predominant poison in the analyzed cases.
• Histopathological findings included pulmonary edema, intra-alveolar hemorrhage, and liver ballooning degeneration.
• Kidney findings showed acute tubular necrosis, while stomach findings revealed partial rugosity loss and mucosal necrosis.
• Stomach congestion, necrosis of the mucosa, epithelial denudation, and inflammation were observed in several cases.

## Graphical Abstract

**Figure:**
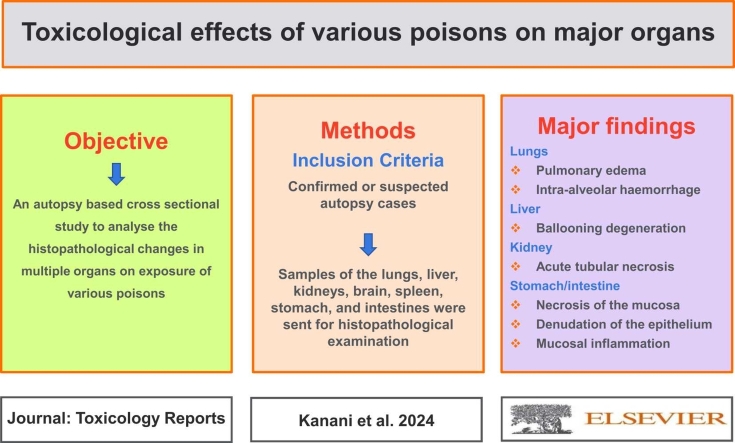

## 1 Introduction

Poisoning occurs when a living organism is exposed to substances capable of causing negative effects on its biological functions. These effects can range from mild irritation to severe harm, potentially leading to death [1]. According to the World Health Organization (WHO), there are three million reported cases of acute poisoning annually, resulting in 220,000 deaths [2]. Developing countries, particularly those relying heavily on agriculture, bear a staggering 99 % of this burden [3]. In developed countries, poisons used in attempts to commit suicide include psychotropic drugs, analgesics, antihistamines, antidepressants, psychoactive drugs, and sedative-hypnotics [4]. In developing countries like India, agricultural poisons, including organophosphates, carbamates, pyrethroids, and aluminum/zinc phosphide, have been identified as the primary culprits. The prevalence of poisoning is worsening due to the constant development of new drugs and chemicals [5]. Unfortunately, pesticides, which are, easily accessible, are misused, contributing to instances of self-harm, especially in times of financial or other stressful situations [6]. The modes of poisoning can vary, ranging from suicide attempts, homicides, to accidental exposures [1]. Deaths from poisoning may occur immediately as a result of respiratory and muscle paralysis, or they can be delayed, leading to complications such as Acute Respiratory Distress Syndrome (ARDS), respiratory paralysis, liver failure, and renal failure.

Determining the precise cause of death in delayed deaths can be challenging, as external appearances and routine internal examinations may not provide sufficient insights. This highlights the crucial role of histopathological examination, which can discover pathological changes in vital organs like the lungs, liver, and kidneys, which are responsible for metabolism and excretion of harmful substance [3]. External and internal postmortem findings play a crucial role in distinguishing acute poisoning cases from other causes of death and histopathological examination helps in differentiating these cases [7]. Histopathology has emerged as a vital tool in assessing the patient's prognosis and facilitating prompt and targeted treatment interventions [8]. This study aimed to contribute to this understanding by analyzing the histopathological findings of various organs in autopsy of poisoning cases.

Our research aimed to identify the specific pattern of histopathological changes in internal organs like liver, kidney, lungs, spleen, and brain. This study is novel as it was conducted to evaluate the effect of different poisons on the stomach and intestine including corrosive poisons, which has not been found in the existing literature.

## 2 Materials and methods

This prospective cross-sectional observational study was carried out in Surat, India, in collaboration with the Department of Forensic Medicine and Toxicology, and the Pathology Department. The primary objective was to establish specific histopathological pattern in internal organs like liver, kidney, lungs, spleen, and brain in poisoning cases. Additionally study was conducted to determine the effects of different types of poisons on stomach and intestine.

### 2.1 Study population

The study included the autopsies of all age groups who had established or suspected history of ingestion of poison, conducted during the timeframe spanning April 2023, to November 2023. Consent was obtained from the relatives before procuring tissue samples from the bodies.

### 2.2 Data collection and analysis

A structured proforma was used to systematically collect socio-demographic details for each poisoning case, containing variables such as age, time of incidence, past medical history, personal history, occupation, income, marital status, and various psycho-social factors associated with suicide (behavior, stressors, substance abuse, psychiatric or physical illness, chronic pain and disability, suicide notes, and previous attempts of suicide, etc.). Individuals were categorized into age groups based on the following criteria: Children: <18 years, Young Adults: 18 years to 30 years, Middle-aged Adults: 31 years to 45 years, Old Adults: Age > 45 years. Thorough external and internal post-mortem examinations were performed and recorded.

### 2.3 Inclusion and exclusion criteria

• **Inclusion criteria**: Cases with a confirmed or suspected history of poisoning and a chemical analysis report were included.
• **Exclusion criteria**: Cases where treatment duration exceeded four weeks, those without proper background information, or those showing signs of decomposition were excluded.

### 2.4 Chemical analysis of toxins

The viscera (stomach, intestine, liver, spleen, kidneys, and blood) were collected and sent to the Forensic Science Laboratory for chemical analysis. The laboratory employed gas chromatography-mass spectrometry (GC-MS), high-performance liquid chromatography (HPLC), steam distillation, volumetric titration, and solvent extraction to detect and quantify the specific toxins present in the tissues. These methods are highly sensitive and capable of identifying even trace amounts of poisons, ensuring accurate toxicological diagnosis.

### 2.5 Histopathological analysis

Tissue samples from the stomach, intestine, liver, spleen, kidneys, brain, and lungs were collected during autopsy and fixed in 10 % formalin saline for 24 hours. The samples underwent a series of processes including dehydration, clearing, impregnation, block formation, microtome sectioning, and staining with hematoxylin and eosin (H&E). The stained slides were examined microscopically to assess histopathological changes. Criteria for histopathological analysis included:

• **Liver**: Presence of ballooning degeneration, sinusoidal dilation, congestion, fatty changes, and necrosis.
• **Kidneys**: Identification of acute tubular necrosis, cloudy degeneration and glomerulosclerosis.
• **Lungs**: Examination for pulmonary edema, intra-alveolar hemorrhage, and acute respiratory distress syndrome (ARDS).
• **Stomach/Intestine**: Assessment of mucosal necrosis, denudation, inflamation, and rugosity.
• **Spleen and Brain**: Evaluation of congestion and any organ-specific pathological changes.

**Assessment tools:** The data were entered into Microsoft Excel software. The SAS OnDemand for Academics software was used for table creation, calculation and statistical analysis. All data (categorical) are expressed as frequencies or percentages. The PROC FREQ procedure was used in the SAS environment.

## 3 Results and discussion

### 3.1 Distribution of poisoning cases

Out of the 52 poisoning cases analysed (Table 1), the majority were due to aluminum phosphide, accounting for 40 cases (76.92 %). Corrosive substances and organophosphorus compounds each were responsible for 5 cases (9.62 %), while psychiatric medicines accounted for 2 cases (3.85 %). Aluminum phosphide is a highly toxic pesticide frequently used to protect stored grains from pests. It is readily available in urban areas and often used for suicide due to its high fatality rate. Most individuals across all types of poisoning died within 24 h of exposure.

**Table 1:** Distribution of poisoning cases with duration of survival (exposure).

Poison	<24 hours	24–72 hours	>72 hours	Number
Corrosive	3	0	2	5 (9.62 %)
Alluminium phosphide	36	3	1	40 (76.92 %)
Organophosphorus	3	1	1	5 (9.62 %)
Psychiatric medicines	1	1	0	2 (3.85 %)
Grand Total	43	5	4	52 (100 %)

Age, sex and marital status wise Distribution of Poisoning Cases (Table 2)

**Table 2:** Demographics distribution of poisoning cases.

Demographics	Number	Percent
Sex		
Female	20	38.46 %
Male	32	61.54 %
Age Group		
Children	3	5.77 %
Middle-aged Adults	12	23.08 %
Old Adults	14	26.92 %
Young Adults	23	44.23 %
Marital Status		
Married	33	63.46 %
Unmarried	19	36.54 %

The distribution of poisoning cases by sex showed that males were more frequently affected, with 32 cases (61.54 %), compared to 20 cases (38.46 %) in females. Poisoning among males aligns with previous research [9], [10], [11]. This preference may be attributed to various factors, including ease of access, lethality, and cultural influence. The age distribution of the poisoning cases showed that the highest incidence was among young adults (23 cases, 44.23 %), followed by old adults (14 cases, 26.92 %). Middle-aged adults accounted for 12 cases (23.08 %), and children represented the smallest group with 3 cases (5.77 %). Young adults, often engaged in educational or work-related activities, may experience stress or pressure from various sources, including academic deadlines, workplace demands, career transitions, financial pressures, and interpersonal issues. In terms of marital status, 33 patients (63.46 %) were married, whereas 19 patients (36.54 %) were unmarried. This finding aligns with previous studies that reported similar trends, with suicide rates among married individuals found to be 57 % in the study by Mohammad et al. (2024), 64 % in the study by Barman and Bairagi (2023), and 75 % in the study by Chaudhari et al. (2022) [9], [12], [13]. Marriage can introduce additional stressors and responsibilities, and issues within the marital relationships or family dynamics can contribute to feelings of distress or hopelessness.

### 3.2 Histopathological changes in various organs

The histopathological examination of organs in poisoning cases reveals significant insights into the pathological processes triggered by different toxic substances. This study highlights the distinct histological changes associated with various poisons.

### 3.3 Lungs

**Fig. 1:**
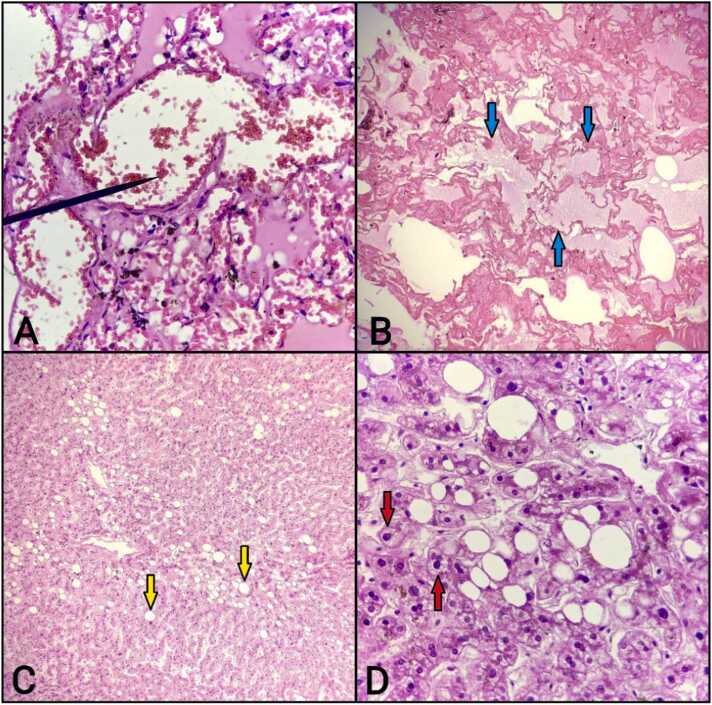
Microscopic section of the lung showing (A) intra-alveolar hemorrhage (black arrow) and (B) pulmonary edema with proteinaceous material in the alveolar spaces (blue arrow). Liver section depicting (C) fatty changes (yellow arrow) and (D) ballooning degeneration of hepatocytes (red arrow).

Histopathological examination [Fig. 1A, 1B] of the lungs revealed pulmonary edema in 29 cases (55.77 %), intra-alveolar hemorrhage in 25 cases (48.08 %), ARDS in 5 cases (9.62 %), inflammatory cell infiltration in 20 cases (38.46 %), and pneumonia in 6 cases (11.54 %) (Table 3). These observations are consistent with the findings of Sawardekar et al. (2020), who reported that 42 % of cases showed intra-alveolar hemorrhage, while 25 % exhibited pulmonary edema [7]. Aluminum phosphide, known for its severe toxicity, was the primary poison associated with these changes, causing pulmonary edema in 48.08 % and intra-alveolar hemorrhage in 36.54 % of the cases. Similarly, the study by Sawardekar et al. found that in rodenticide poisoning, pulmonary edema was observed in 67 % of cases, and intra-alveolar hemorrhage in 17 % of cases [7].These findings highlight the toxic effects of aluminum phosphide on respiratory tissues, leading to significant edema and hemorrhage due to capillary damage and increased vascular permeability. Inflammatory cell infiltration and pneumonia, though less common, were also noted, suggesting that severe cases of poisoning may predispose patients to secondary infections and inflammatory responses.

**Table 3:** Histopathological changes in lungs.

Poison	Pulmonary edema	Intraalveolar hemorrhage	ARDS	Inflammatory cell infiltration	Pneumonia
Corrosive	0 (0 %)	1 (1.92 %)	1 (1.92 %)	3 (5.77 %)	1 (1.92 %)
Alluminium phosphide	25 (48.08 %)	19 (36.54 %)	4 (7.69 %)	15 (28.85 %)	4 (7.69 %)
Organophosphorus	2 (3.85 %)	3 (5.77 %)	0 (0 %)	1 (1.92 %)	1 (1.92 %)
Psychiatric medicines	2 (3.85 %)	2 (3.85 %)	0 (0 %)	1 (1.92 %)	0 (0 %)
Grand Total	29 (55.77 %)	25 (48.08 %)	5 (9.62 %)	20 (38.46 %)	6 (11.54 %)

### 3.4 Liver

The liver, a critical organ for detoxification, showed marked histopathological changes [Fig. 1C,1D] across various poisoning cases. Ballooning degeneration and congestion were predominant, particularly in cases involving aluminum phosphide, which accounted for 48.08 % and 42.31 %, respectively, followed by fatty changes at 32.69 % (Table 4). Previous studies conducted by Pednekar et al., Siddesh and Dey, Sutay and Tirpude, Sawardekar et al., and Adhikari et al. also demonstrated that fatty changes and congestion were predominant findings in poisoning-related liver autopsies, aligning with the results of the present study [1], [3], [7], [8], [14]. The high incidence of fatty changes and necrosis further underscores the liver's vulnerability to toxic insults. These findings are consistent with the liver's role in metabolizing toxins, where toxic metabolites can induce hepatocellular damage, steatosis, and ischemic changes due to impaired blood flow.

**Table 4:** Histopathological changes in liver.

Poison	Ballooning degeneration	Sinusoidal dilation	Congestion	Necrosis	Fatty changes
Corrosive	3 (5.77 %)	1 (1.92 %)	2 (3.85 %)	1 (1.92 %)	2 (3.85 %)
Alluminium phosphide	25 (48.08 %)	6 (11.54 %)	22 (42.31 %)	8 (15.38 %)	17 (32.69 %)
Organophosphorus	3 (5.77 %)	0 (0 %)	0 (0 %)	1 (1.92 %)	3 (5.77 %)
Psychiatric medicines	0 (0 %)	0 (0 %)	1 (1.92 %)	0 (0 %)	1 (1.92 %)
Grand Total	31 (59.62 %)	7 (13.46 %)	25 (48.08 %)	10 (19.23 %)	23 (44.23 %)

### 3.5 Kidneys

**Fig. 2:**
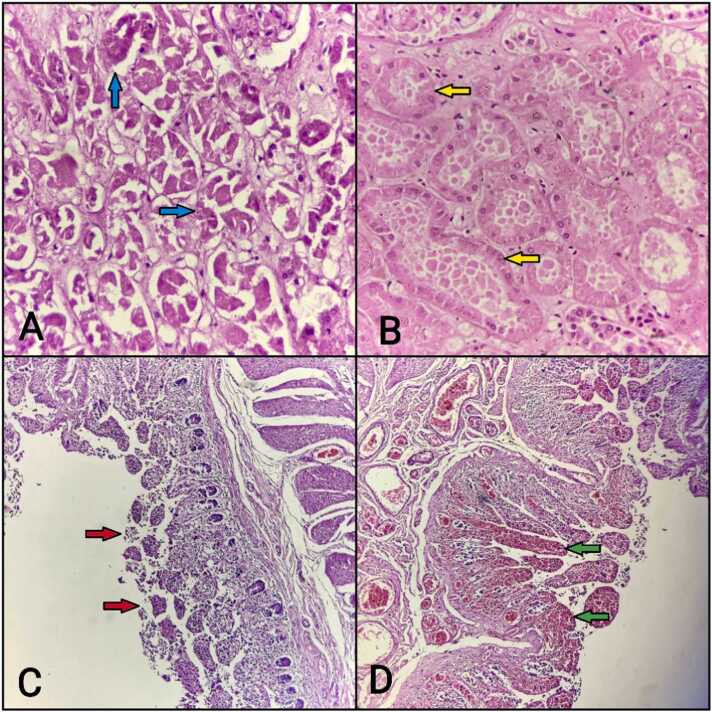
Microscopic section of the kidney showing (A) acute tubular necrosis with coagulative necrosis of renal tubules (blue arrow) and (B) cloudy degeneration of renal tubules (yellow arrow). (C) Denudation of the epithelium (red arrow) and (D) intestinal hemorrhage (green arrow).

In the kidneys (Table 5), acute tubular necrosis [Fig. 2A] was observed in 27 cases (51.92 %), congestion in 21 cases (40.38 %), cloudy degeneration [Fig. 2B] in 29 cases (55.77 %), and glomerulosclerosis in 10 cases (19.23 %). These findings align with previous studies, where Siddesh and Dey reported 100 % congestion and 85 % acute tubular necrosis, while Sawardekar et al. observed 44.2 % acute tubular necrosis and 50 % cloudy degeneration [3], [7]. Aluminum phosphide poisoning showed a high prevalence of these changes, indicating its nephrotoxic potential. Acute tubular necrosis, observed in 40.38 % of aluminum phosphide cases, points to direct tubular damage and ischemia, which are well-documented effects of severe systemic toxicity. Cloudy degeneration and congestion reflect the kidneys' response to oxidative stress and disrupted microcirculation.

**Table 5:** Histopathological changes in kidneys.

Poison	Acute tubular necrosis	Congestion	Cloudy degeneration	Glomerulosclerosis
Corrosive	2 (3.85 %)	0 (0 %)	4 (7.69 %)	1 (1.92 %)
Alluminium phosphide	21 (40.38 %)	20 (38.46 %)	22 (42.31 %)	7 (13.46 %)
Organophosphorus	2 (3.85 %)	1 (1.92 %)	3 (5.77 %)	2 (3.85 %)
Psychiatric medicines	2 (3.85 %)	0 (0 %)	0 (0 %)	0 (0 %)
Grand Total	27 (51.92 %)	21 (40.38 %)	29 (55.77 %)	10 (19.23 %)

### 3.6 Brain

Histopathological changes in the brain included congestion in 52 cases (100 %) and hemorrhage in 2 cases (3.85 %) (Table 6). Aluminum phosphide was implicated in the majority of these cases (40 cases, 76.92 %). The universal finding of congestion indicates cerebral vascular involvement in systemic poisoning. The brain’s susceptibility to toxins is linked to compromised blood-brain barrier integrity and resultant vascular congestion, which can exacerbate cerebral hypoxia and neuronal injury.

**Table 6:** Histopathological changes in brain.

Poison	Congestion	Hemorrhage
Corrosive	5 (9.62 %)	0 (0 %)
Alluminium phosphide	40 (76.92 %)	1 (1.92 %)
Organophosphorus	5 (9.62 %)	1 (1.92 %)
Psychiatric medicines	2 (3.85 %)	0 (0 %)
Grand Total	52 (100 %)	2 (3.85 %)

### 3.7 Spleen

The spleen (Table 7) showed congestion in all 52 cases (100 %), with aluminum phosphide being the predominant poison (40 cases, 76.92 %). The spleen’s role in filtering blood makes it particularly susceptible to congestion due to systemic toxins, reflecting impaired venous return and increased splenic sequestration of blood.

**Table 7:** Histopathological changes in spleen.

Poison	Congestion
Corrosive	5 (9.62 %)
Alluminium phosphide	40 (76.92 %)
Organophosphorus	5 (9.62 %)
Psychiatric medicines	2 (3.85 %)
Grand Total	52 (100 %)

### 3.8 Stomach

Morphological examination (Table 8) of the stomach revealed rugosity partially lost in 42 cases (80.77 %) and congestion in 27 cases (51.92 %). Aluminum phosphide was again the main contributor, causing rugosity loss in 32 cases (61.54 %) and congestion in 21 cases (40.38 %).

**Table 8:** Morphological findings in stomach.

Poison	Rugosity partially lost	Congestion
Corrosive	4 (7.69 %)	4 (7.69 %)
Alluminium phosphide	32 (61.54 %)	21 (40.38 %)
Organophosphorus	4 (7.69 %)	2 (3.85 %)
Psychiatric medicines	2 (3.85 %)	0 (0 %)
Grand Total	42 (80.77 %)	27 (51.92 %)

Histopathological changes [Fig. 2C] in the stomach included necrosis of the mucosa in 16 cases (30.77 %), congestion in 37 cases (71.15 %), denudation of the epithelium in 25 cases (48.08 %), and mucosal inflammation in 25 cases (48.08 %) (Table 9). Our study outcomes are in concordance with previously reported data [15], [16], [17]. Aluminum phosphide was the leading cause of these changes, particularly causing congestion (29 cases, 55.77 %) and denudation of the epithelium (27 cases, 51.92 %). These changes reflect direct corrosive effects of ingested toxins on gastric mucosa, leading to inflammation, ulceration, and necrosis. The high incidence of mucosal damage underscores the importance of early intervention in cases of ingested poisons to prevent severe gastrointestinal complications.

**Table 9:** Histopathological changes in stomach.

Poison	Necrosis of mucosa	Congestion	Denudation of epithelium	Mucosal inflamation
Corrosive	3 (5.77 %)	2 (3.85 %)	3 (5.77 %)	2 (3.85 %)
Alluminium phosphide	12 (23.08 %)	29 (55.77 %)	27 (51.92 %)	20 (38.46 %)
Organophosphorus	0 (0 %)	4 (7.69 %)	4 (7.69 %)	3 (5.77 %)
Psychiatric medicines	1 (1.92 %)	2 (3.85 %)	2 (3.85 %)	0 (0 %)
Grand Total	16 (30.77 %)	37 (71.15 %)	25 (48.08 %)	25 (48.08 %)

### 3.9 Intestine

Histopathological examination [Fig. 2D] of the intestine showed inflammatory cell infiltration in 28 cases (53.85 %), congestion in 34 cases (65.38 %), and denudation in 26 cases (50 %) (Table 10). Aluminum phosphide was the most frequent cause, particularly causing congestion (28 cases, 53.85 %) and denudation (31 cases, 59.62 %). These findings indicate that the intestines, similar to the stomach, are highly susceptible to direct toxic effects, leading to widespread inflammation and mucosal damage. The presence of denudation and congestion underscores the potential for severe gastrointestinal bleeding and compromised nutrient absorption.

**Table 10:** Histopathological changes in intestine.

Poison	Inflamatory cell infiltraion	Congestion	Denudation
Corrosive	2 (3.85 %)	3 (5.77 %)	3 (5.77 %)
Alluminium phosphide	22 (42.31 %)	28 (53.85 %)	31 (59.62 %)
Organophosphorus	3 (5.77 %)	2 (3.85 %)	4 (7.69 %)
Psychiatric medicines	1 (1.92 %)	1 (1.92 %)	2 (3.85 %)
Grand Total	28 (53.85 %)	34 (65.38 %)	26 (50 %)

These findings have several important implications. The detailed histopathological patterns identified in this study can aid clinicians in the early diagnosis and management of poisoning cases. Understanding the specific organ damage associated with different poisons can lead to more accurate diagnoses and tailored treatment plans, potentially improving patient outcomes. The high incidence of poisoning due to aluminum phosphide, particularly in suicidal cases, underscores the need for stringent regulatory measures and public health interventions. This includes controlling the availability of such highly toxic substances and increasing awareness about their dangers. Public health campaigns and mental health support services could be crucial in reducing the incidence of poison-related suicides. The specific tissue changes documented can serve as valuable reference points for forensic pathologists, helping to distinguish poisoning from other potential causes of death.

## 4 Conclusion

The correlation between histopathological changes and different types of poisoning provides valuable insights into the mechanisms of organ damage in poisoning cases. Aluminum phosphide emerged as the most commonly involved poison, causing severe histopathological changes across all examined organs. The findings of this study underscore the critical role of histopathological examination in understanding the extent and nature of organ damage in poisoning cases, facilitating accurate diagnosis and targeted therapeutic interventions. Continued research and documentation of histopathological changes in poisoning cases are essential for advancing our understanding of toxicopathology and improving patient outcomes.

## Ethics approval and consent to participate

Ethical approval and the need for informed consent were waived for this study as it utilized deidentified data and autopsy specimens. All patient data used in this study were anonymized to protect patient privacy and confidentiality.

## Consent for publication

All the authors approved the final version of this research and consented to publication.

## Funding

This research did not receive any specific grant from funding agencies in the public, commercial, or not-for-profit sectors.

## CRediT authorship contribution statement

**Swati Mesuriya:** Writing – review & editing, Writing – original draft, Supervision. **Sudha Jain:** Writing – review & editing, Writing – original draft, Investigation, Data curation. **Mohammed Iliyas Sheikh:** Writing – review & editing, Writing – original draft, Visualization, Validation, Investigation, Conceptualization. **Jayeshkumar Kanani:** Writing – review & editing, Writing – original draft, Visualization, Validation, Supervision, Software, Resources, Methodology, Investigation, Formal analysis, Data curation, Conceptualization.

## Declaration of Competing Interest

The authors declare that they have no known competing financial interests or personal relationships that could have appeared to influence the work reported in this paper.

## Data Availability

Data will be made available on request.
